# Intestinal Capillariasis, Western Mindanao, the Philippines

**DOI:** 10.3201/eid1604.080483

**Published:** 2010-04

**Authors:** Vicente Y. Belizario, Francis Isidore G. Totañes, Winifreda U. de Leon, Julius R. Migriño, Lino Y. Macasaet

**Affiliations:** University of the Philippines–National Institutes of Health, Manila, the Philippines (V.Y. Belizario Jr, F.I.G. Totañes, J.R. Migriño Jr); University of the Philippines College of Public Health, Manila (W.U. de Leon); National Center for Disease Prevention and Control, Manila (L.Y. Macasaet)

**Keywords:** Capillariasis, foodborne helminths, soil-transmitted helminths, parasitology, parasites, the Philippines, letter

**To the Editor:** Capillariasis is caused by the foodborne nematode *Capillaria philippinensis*. Infection causes severe diarrhea and protein loss resulting in dehydration, cachexia, and eventually death. Infected patients may also have borborygmi, abdominal pain, weight loss, anorexia, vomiting, and bipedal edema (*1*).

*C*. *philippinensis* was first reported in 1963 in Bacarra, Ilocos Norte Province in the northern Philippines (*2*). Since then, additional endemic foci of *C*. *philippinensis* have been identified. The most recent focus is in Monkayo, Compostela Valley, in the southern Philippines (*3*). In the past several years, suspected unconfirmed cases have been reported from Zamboanga del Norte Province in western Mindanao. In 1999, an epidemic of gastroenteritis in Piñan Municipality was reported; it resulted in 42 deaths. The schistosomiasis team of the Department of Health Regional Office conducted stool examinations and suspected the presence of *Capillaria* ova in symptomatic patients (*4*). In November 2007, several deaths caused by chronic diarrhea were reported in Siayan Municipality. These deaths were attributed to capillariasis, but their cause was never confirmed (*5*).

In February 2008, we obtained 205 stool specimens from residents of Katipunan who had a history of diarrhea of >2 weeks duration and abdominal disturbance. These samples were processed by using the formalin–ether concentration technique (*6*) and examined by expert microscopists. One hundred fifty-one (73.3%) persons were infected with >1 organism; 67 (32.5%) had 1 parasitic infection and 84 (40.8%) had multiple parasitic infections. Ninety-one (44.2%) persons had >1 soil-transmitted helminth infection, and 93 (45.2%) had >1 protozoan infection. Ten (4.9%) persons were confirmed to have *Capillaria* infections. The distribution of organisms observed is shown in the Table.

**Table:** Parasites detected in Katipunan, Zamboanga del Norte, the Philippines, February 2008*

Parasite	No. (%)
*Trichuris trichiura*	64 (31.1)
*Entamoeba coli*	49 (23.8)
*Ascaris lumbricoides*	46 (22.3)
*Endolimax nana*	14 (19.9)
Hookworm	34 (16.5)
*Blastocystis hominis*	21 (10.2)
*Giardia lamblia*	19 (0.2)
*Entamoeba histolytica*	14 (6.8)
*Capillaria philippinensis*	10 (4.9)

*A total of 205 parasites were detected by using the formalin–ether concentration technique.

Among the 10 persons who had capillariasis, 8 were from Barangay (smallest adminstrative region) Matam, 1 from Barangay Dabiak, and 1 from Barangay Carupay, a nearby barangay. Six cases were in male patients and 4 were in female patients. Ages of infected persons ranged from 5 to 54 years (mean 29.2 years, SD 17.1 years). Three of the reported case-patients (a 5-year-old boy, an 8-year old boy, and a 48-year-old woman) were from the same household.

A total of 24 persons in Katipunan were interviewed regarding history of capillariasis and their eating habits. Fourteen residents reported having eaten kinilaw (raw freshwater fish soaked in vinegar and garnished with salt, ginger, and lime). Seven of the persons interviewed had a diagnosis of capillariasis, and 6 had >1 relative with a diagnosis of capillariasis. All of the previously diagnosed case-patients were treated with albendazole (400 mg tablets). Most patients were instructed to take 1 tablet 1×/day for 20 days; others were instructed to take 1 tablet 2×/day for 5 or 10 days.

The drug of choice for treating patients with capillariasis is mebendazole, 200 mg 2×/day for 20–30 days. An alternative treatment is albendazole, 400 mg 1×/day for 10 days (*7*,*8*). Variations in the treatment regimen used for patients with capillariasis at the study site suggest a need to train health professionals on the diagnosis, treatment, and follow-up of cases, and on disease prevention and control. Guidelines on proper laboratory techniques for diagnosis of capillariasis; treatment protocols and supportive measures; and protocols for detection, follow-up, and treatment for relapse cases must be developed.

Rates of infection with protozoans and soil-transmitted helminths at the study site are high, which indicate fecal contamination of food and water. A review of records from the Katipunan rural health unit indicated that 76% of households in this municipality have access to toilets. Only 11% of households have water connections (level III). Fifty-seven percent of households have access to communal faucets (level II), and 31% have access only to rivers or springs (*9*). Therefore, increased access to toilets and safe water is needed. Local ordinances concerning ownership and use of toilets must be strictly enforced, and evaluation and rehabilitation of existing toilet and water systems must be conducted.

In spite of efforts concerning information, education, and communication on capillariasis, many residents continue to eat raw or poorly cooked freshwater fish. Concurrent infection among household members, including those in younger age groups, was observed in this study. These findings result from the fact that consumption of kinilaw has become widely accepted and is consumed as a viand (choice food) by families. Thus, information, education, and communication campaigns must be intensified. A promising approach is through collaboration with other agencies. For example, the Department of Education in the Philippines may become involved in dissemination of information on capillariasis to students and in early detection and treatment of infected school children.
